# Generative listening leading to generative action to enhance interdisciplinary connections in a specialized referral hospital setting

**DOI:** 10.1016/j.clinsp.2026.100886

**Published:** 2026-03-04

**Authors:** Alfredo Jose Mansur, Orival de Freitas Filho, Cicero Piva de Albuquerque, Brigitte Feiner de Mello, Leila Suemi Harima Letaif, Edivaldo Massazo Utiyama

**Affiliations:** a Faculdade de Medicina da Universidade de São Paulo (FMUSP), São Paulo, SP, Brazil; b Instituto do Coração, Hospital das Clínicas, Faculdade de Medicina, Universidade de São Paulo (InCor-HCFMUSP), São Paulo, SP, Brazil

**Keywords:** Organizational culture, Corporate culture, Clinical governance, Institutional management teams, Professional practice

## Abstract

**Objectives:**

To contribute to the organizational culture and interdisciplinary connections to surpass frontiers created by evolving specialization that put professionals as well as processes apart. In addition, we preserved and stimulated local established professional skills.

**Methods:**

The authors worked by listening to organizational demands as well as demands of colleagues at work in a network organizational non-mandatory working weekly forums through videoconferences. Forums were devoted to specific scenarios, such as surgical center facilities, outpatient clinics, hybrid rooms, clinical directors, multidisciplinary leadership, intensive care units’ leadership, and institutional life. Mandatory (by local regulation) subcommittee forums occurred every two weeks. Participants of the meetings were in their working place. Every leadership would be present at most in two forums each week; participation might rotate between local leaderships.

**Results:**

200 interdisciplinary nonmandatory working forums were carried out in 2024; number of invites ranged between 52 (hybrid room) and 131 (clinical units’ leadership), participations ranged between 718 (hybrid room) and 791 (clinical units leadership) and mean participation ranged between 17.4 (range 11‒25; standard deviation 3.21) and 20.1 (range 11‒27; standard deviation 3.40). Hundred six mandatory forums were carried out in 2024; the number of invites ranged between 10 (subcommittee of pharmacology) and 19 (review of mortality); mean participation ranged between 9 (range 5‒12; standard deviation 2.16) and 11.7 (range 6‒15; standard deviation 2.24). Specific practical initiatives were born from these forums.

**Conclusions:**

The authors consider that interdisciplinary connectivity was participative to a shared understanding of processes and associated requirements.

## Highlights

• Surpassing organizational frontiers through listening in regular harmonization forums.
• Fostering interdisciplinary connections through listening in harmonization forums.
• Harmonization working forums as a strategy for regular listening.
• Harmonization working forums to enhance interdisciplinary connections.
• Interdisciplinary connectivity to understanding organizational processes.

## Introduction

Referral hospitals are complex work environments encompassing activities with both healthy and sick people and their families provided by a large staff of healthcare professionals who work to the best of their abilities in the multifaceted process of care and recovery of sick people. Processes involved in the care of patients are broad in scope, inherently complex by their nature, and require different levels of expertise permeated in the organization's culture. Unsurprisingly, to bring together skill, strength, and occasional weaknesses, harmoniously, in order to work out efficiently and safely is an everyday challenge for these institutions. Organization culture has been defined in different ways, previously quoted.^1^ “invented, discovered, or developed by a given group as it learns to cope with its problems of external adaptation and internal integration ‒ that has worked well enough to be considered valid and, therefore, to be taught to new members as the correct way to perceive, think, and feel in relation to those problems”.^1^ In addition, a specific hospital may be connected to a network of care of different levels of complexity, either within the city or somewhere else outside of the state or country. Thus, the hospital work includes a combination of receiving patients from and referring patients back to primary or secondary levels of care.

The authors have been working for (six) years in the clinical governance of a large university hospital in São Paulo, Brazil. Gradually, the authors had the opportunity to develop some local communication channels in collaboration with hospital teams, inspired by central supervision and coordination. The authors started a work program devised to be a contribution to the institutional mission and governance (in accordance with institutional values, goals and strategies), taking into account the human factor of healthcare professionals (basic underlying assumptions, which are the sometimes unconscious, taken-for-granted beliefs, perceptions, thoughts and feelings), operation (structure and processes)^1^ aiming, on the basis the available structures and staff, improving results in performance, either in quality or in production numbers, through fostering discovery between different teams, smooth and collaborative relationships and empowerment of patients, families and health staff in their care.

Large institutions may evolve with departmentalization and compartmentalization.^2^ due to the complexity of health care. In the event that the number of departments might increase, they may put people apart, far away from each other. Such distancing may make connections sometimes protracted, less timely, or more bureaucratic and undermine the ability of professional exchange in operational fields and in managing areas, as well as in caring for patients. The gap caused by “departmentalization” creates “systemic” limits to multidisciplinary communication and teamwor;k.^2^ “compartments” may evolve to become “islands” within the organization, with poor communication with other compartments.^2^ Systemic thinking may be one strategy for surpassing these limitations.^3^

Sometimes processes may suffer the influence of naturalistic culture or sciences of nature^4^ in becoming scientific and supposedly without intentionality, that is, impersonal, to the point that it may become difficult to identify responsibilities. In complex processes that lead to distant effects and unintentional effects, neither the author may be easily identified, nor the responsibility can be easily estimated. The contribution of several participants in a complex action may also be very difficult to establish.^4^ Some steps of the creative work were interpreted, while there may be criticism about the mathematical modeling contends to have chaotic dynamics that modulate to rigidity in a space of learning, adaptation, and innovation through connectivity.^5^ In addition, there may be a human dimension to many technological operations.^6^ mainly in health care directed to sick people. Furthermore, there are intangible and tangible themes in work environments; a synthesis of categorization into intangible and tangible themes was developed after applying commonly used questionnaires.^7^ Intangible themes were commitment, trust, psychological safety, power, support, communication openness, blame and shame, morals and valuing ethics, and cohesion. Tangible themes were leadership, communication system, teamwork, training and development, organizational structures and processes, employee and job attributes, and patient orientation.^7^

The goals of our work have been to address the ethos of the organization, taking into consideration tangible and intangible dimensions of culture as a member of the staff at first by observation.^8^ conveying staffs together in connectivity as a value.^5^ next to each other, listening, seeing each other, discovering inconsistencies, diagnosing and pursue measures that might fulfill the needs of the patients, in accordance with institutional governance and mission to reach a systemic engagement of the staff.^3^

## Materials and methods

### Setting

The medical school and University Hospital (FMUSP ‒ Hospital das Clínicas Academic System) include 2400 hospital beds in addition to a large outpatient center with more than 1.5 million outpatient consultations per year. This medical complex includes specialized hospitals dedicated to several medical specialties in a central building, and separate buildings for orthopedics, psychiatry, pediatrics, lung and heart diseases, and rehabilitation. Our work was developed in the Heart Institute of the hospital complex (Heart Institute, InCor), an independent building with 503 hospital beds and active outpatient clinics in the year 2024, that performed 165,475 medical consultations, in addition to surgeries (5731) and different modalities of heart catheterization and percutaneous interventions (13,983). House staff is constituted of approximately 3637 healthcare professionals (June 2025).

### Principles

The authors cultivated the following precepts as essential to the project: a) Low profile leadership; b) Soft skills in dealing with the staff; c) Not restricted to a single and personal leadership; d) Shared and rotating leadership in different multiple activities; e) Multidisciplinary and interdisciplinary work; f) Agglutination of inputs received from our coordination guidance (from the leadership of the Hospital complex and from the leadership of the Heart Institute) as well as retrieved from the colleagues in everyday interdisciplinary work; *Dasein* (in practical terms) – being present and feeling stimulated to approach the professional and human experience in an in-depth and rewarding attitude, in the health care environment and according to the demands. Staff were led to convene next to each other, listening, seeing each other, discovering inconsistencies, understanding and improving operations according to the needs of the patients, as well as proposing updates and planning for the future in accordance with institutional governance and mission.

### Steps definitions

**Fig. 1:**
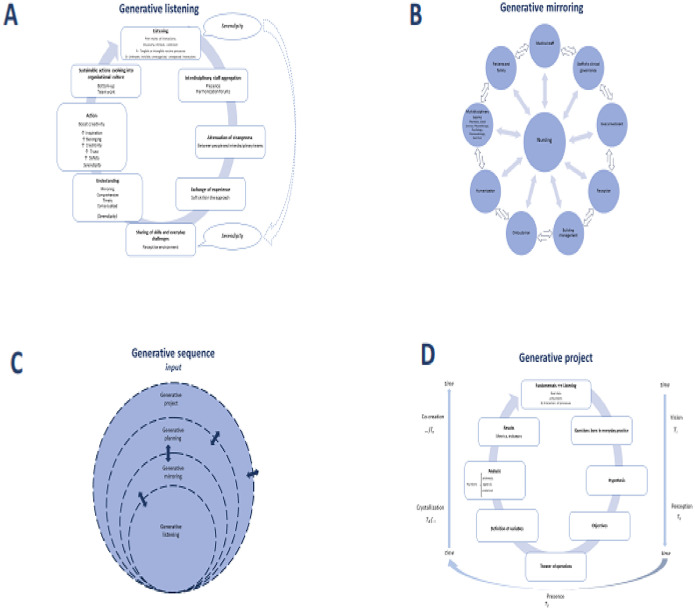
(A) Steps of generative listening to build sustainable performance. (B) Mirroring as a strategy to build empathetic environment and systemic thinking. (C) Generative sequence of integrating facts and data. (D) Generative project taking time as a key dimension (adapted from Scharmer CO. O essencial da teoria U: princípios e aplicações fundamentais. Curitiba: Editora Voo, 2020).^15.^

The authors followed the steps: a) Listening; b) Mirroring; c) Serendipity experiences; d) Planning (schematization); d) Projects (praxis) (Fig. 1).

#### Listening

Actively listening to contributions of colleagues in everyday practice, evolving gradually and openly to a sustained receptive state of mind by the interdisciplinary staff discussing practical issues of specific work scenarios, connecting processes and professionals in different forums, preventing inconsistencies and aiming to improve the process of healthcare. Listening was particularly useful to retrieve intangible or unnoted dimensions.^1^ of everyday challenges and evolved in several steps to reach sustainable performance and culture (Fig. 1A). The contributions were registered in such a way to lead to developments and upgrades in the solution of everyday challenges.

Listening was facilitated by regular meetings of interdisciplinary faculty (devoted to surgical center staff, outpatient clinics, hybrid room, clinical directors, difficult situations in clinical practice, multidisciplinary staff, intensive care units’ leadership, and an institutional level). In each meeting: a) The authors brought information of the highest leadership of the hospital complex and of local leadership; b) Revised the work of the past week; c) The authors opened for participants to bring details that they deserve interdisciplinary discussion; d) Whenever pertinent the authors stimulated exchange of interdisciplinary experience or demands in specific issues; e) Tasks or themes to be addressed in next meeting or to developed in practical issues.

#### Serendipity

Was captured when participants told details of everyday practice, brought unknown or unnoticed details or lack of continuity between processes. Through listening in a receptive state of mind, on studying the practice of everyday patient care in the forums and other exchange experience the authors perceived the possibility of obtaining by chance valuable insights not sought for. In addition, and maybe as important, the discovery in the working groups of intangibles, unintended, previously unrecognized, worked out or addressed events was a distinctive feature of our working forums and blended in the generative sequence (Fig. 1A).

#### Mirroring

Our human and technical-scientific work processes may be considered as a mirror to the ethos of our institutional mission; the authors may be able to see ourselves and each other through this mirror, patients and healthcare professionals, in an evolving process in which the central position rotates according to the specific therapeutic step, interchanging over time, and moving towards the mission which the authors are all devoted to (Fig. 1B).

#### Planning

Planning was built with people (local resources, with strengths and fragilities), material resources (limitations, logistics, etc.), and aiming results (key performance indicators) within a collective construction (Fig. 1C). Specific tasks were conducted by the specific professional group.

#### Projects

Specific step of the development, taking the specific goals developed after evaluating the experiences of patients and health professionals (Fig. 1D).

### Time-related issues

Usually, time is not depicted as it is recognized by the perceptions of staff and patients. Translation into numbers may not be representative of the consciousness of time associated with the human dimensions involved. The balance between anxiety and procrastination of each step dealing with healthcare is challenging, since usually everything is expected to be executed as quickly as possible. Meetings were planned not to exceed 60 min.

### Intervention

The authors developed, after 2020, working groups that we nominated *harmonization forums,* which were devoted to specific and broad healthcare activities in the hospital in weekly or biweekly intervals, devised to attenuate the compartmentalization that might put health care professionals apart and develop a shared systemic view of processes. The agenda was planned for each working forum based on day-to-day needs. It is worth noting that the intervention was established during the COVID-19 epidemics, a time when it was particularly propitious for the staff to develop the abilities necessary to perform virtual conference calls.

The harmonization forums were working meetings that joined weekly staff leaders of specific groups of care either by video or in person. New interdisciplinary working groups were born, created after topics emerged from the harmonization forums. Mandatory subcommittees required by local health regulations had meetings every two weeks.

Protocol of harmonization forums was: a) The authors delivered strategic information of the highest leadership of the hospital complex and of local leadership; b) Revised the work of the past week; c) The authors open for participants stimulating to bring details or data that might deserve interdisciplinary discussion; d) Whenever pertinent the authors stimulated exchange of interdisciplinary experience or demands; e) Tasks or themes to be addressed in next meeting or to developed in practical issues. Participants of the forums didn´t leave their working position for participation in the forums, just accommodated according totheir own schedules.

1. Specific forums: a) Surgical center staff and associated professionals; b) Outpatient clinics staff and associated professionals, including clinical pathology and imaging staff; c) Hybrid room staff and associated professionals; d) Directors of clinical cardiology and pulmonology divisions and associated staff; e) Multidisciplinary directors and staff, including nurses, physiotherapists, psychologists, social workers, pharmacists, ombudswoman, and others as needed and invited; f) Leaders of intensive care units and associated staff; g) Live broadcast to the hospital intranet in association with the chief administration officer open to the entire hospital staff (3637 collaborators, June 2025) every week on Fridays 11:30‒12:00 h.
2. Interdisciplinary working groups derived from the previous forums – a) Sustainability; b) Serendipity; c) Difficult situations in the care of ward patients; d) Technology of information.
3. Mandatory interdisciplinary subcommittees (connected with a central committee of the hospital complex) – a) Committee devoted to care of documentary institutional licenses, audits and supervision; b) Committee devoted to pharmacy issues; c) Committee devoted to therapy with blood and blood-derived constituents; d) Hospital mortality committee with the mission of analyzing every in-hospital death.

### Ethical aspects

Since the manuscript examines only the management strategies developed during the healthcare work without the direct involvement of patients, the IRB review was not considered necessary. This project was an initiative to restructure the organization into interdisciplinary groups and empower them. As it does not affect clinical decisions of care, ethics committee review and approval were not needed.

## Results

The authors report the activities in the three categories of the working forums.

I) Weekly harmonization forums of specific meetings. The authors selected the activities of the year 2024, with the frequency of working meetings, of participations (Table 1) and selected three main themes studied and worked in each forum (Table 2). Some themes might be examined in more than one forum and may recur to be studied in more depth according to the operational needs.

**Table 1:** Frequency of specific forums related to the frequency of participations (2024).

Forum	Frequency of forums	Frequency of invites per forum	Frequency of participations	Mean (min‒max; SD) of participations
Clinical units’ leaderships	41	131	791	19.2 (9‒27; 3.64)
Intensive care units’ leaderships	36	80	865	19.9 (13‒31; 3.87)
Surgical center staff	43	62	738	20.1 (11‒27; 3.40)
Hybrid operating room staff	42	52	718	17.4 (11‒25; 3.21)
Multiprofessional leaderships	38	59	748	19.6(12‒26; 2.87)

min, Minimum; max, Maximum; SD, Standard Deviation.

**Table 2:** Selected topics covered in the harmonization forums and associated subcommittees and working groups.

Forums	Selected topics
Surgical center staff	Review of surgical schedule and activity of previous week
	Review of emergency surgical operations of the previous week, including heart and lung transplantations
	Surgical records
Hybrid room staff	Review of surgical schedules and activity of previous week
	Optimization of interdisciplinary team performance in hybrid room
	Outcomes of patients submitted to transcatheter aortic valve implantation
Directors of clinical units	Counter-reference of patients to other medical resources
	Registering of therapeutic planning
	Critical review of practices and infection control
Directors of multiprofessional units	Pharmacists contribution to the treatment of patients
	Counter-reference of outpatients
	Social issues in the transition of care
Leaders of intensive units	Bedside procedures registering
	Prescriptions and vital controls in shared electronic forms
	Internet connections of medical equipment
Institutional live calls	Information of decisions of the board of Directors
	Interview of an invited colleague
	Introducing new colleagues to
Subcommittee of review of mortality data	Review of each clinical case of deceased patients
	Corrections of death certificates
	Study of potential side effects of medical therapy
Subcommittee of transfusion medicine	Harmonization of the flow of blood resources for transfusion
	Integration of computer assisted systems for controlling the therapies
	Transfusional reactions
Subcommittee of pharmacology	Polypharmacy
	Transition of care to outpatients’ facilities after hospital discharge
	Integration of drug dispensation processes
Subcommittee of auditing medical records	Findings in auditing electronic medical records
	Research in computational linguistics for auditing
	Interdisciplinary work
Critical situations in the care of patients	Study of situations that were perceived as uncomfortable by the staff
	Study of difficult situations of patients’ health conditions as well as families
	Support health care professionals after potentially traumatic experience
Working group of serendipity	Surgical flow of patients
	Harmonization of prescription in different intensive unit’s care
	Internal transition of care
Working group of medical prescriptions	Monitoring of antibiotic prescriptions
	Optimization of integrating medical prescriptions, pharmacists, nurses
	Time issues related to prescription and delivery of therapy

The frequent working forums were associated with the ability of staff interactions, listening, addressing sensitive issues in connection with processes to the point of being able to make pragmatic decisions, preventing internal conflicts, and increasing objectivity, diagnoses, and decisions over time. Adherence to the initiative was considered adequate.

I) Operational weekly interdisciplinary working teams derived from the harmonization forums ‒ the working groups were developed inspired by institutional needs and themes discussed in the different forums and was ran be interdisciplinary teams without the need for leadership of the processes. Inspiration and creativity led to routine discoveries of redundancy or repetition of work, or inconsistencies of communication exchange in some processes.
II) Mandatory subcommittees demanded by local regulation every two weeks. The authors selected the activities of the year 2024, with the frequency of working meetings, of participations (Table 3), and main themes studied and worked.

**Table 3:** Frequency of specific mandatory subcommittees forums related to the frequency of participations (2024).

Subcommittee forums	Frequency of forums	Frequency of invites per forum	Frequency of participations	Mean (min‒max; SD) of participations
Review of mortality data	19	24	134	7.4 (5‒10; 1.3)
Transfusion medicine	15	24	134	8.9 (6‒15; 2.31)
Pharmacology	10	23	90	9 (5‒12; 2.16)
Auditing of medical records	18	22	211	11.7 (6‒15; 2.24)

min, Minimum; max, Maximum; SD, Standard Deviation.

These traditional mandatory subcommittees' performance has been previously more related to strict administrative or strict technical issues. The authors have been working in create an environment of careful judgement of processes and process interactions on an institutional and systemic view, and not just in specific terms or professionals. Research processes have been stimulated, for instance, in electronic medical records audits with computational linguistics, studies of health care in relation to mortality, and more recently, studies of dialysis staff were started.

I) Outpatient clinics staff and associated professionals, including clinical pathology and imaging staffs were started in February 2025

## Discussion

The strategy the authors followed was aimed to surpass compartmentalization that might make healthcare less fluent. The authors were happy to observe that many colleagues were adherent and regular participants of the forums. The videoconferences made it possible to join a number of colleagues that would not be otherwise possible. One of the best aspects of our working forums was the receptive environment that created encouragement by the careful listening of the day-to-day experiences of staff, their difficulties and questions, and by sharing experience in an interdisciplinary connection of a diversity of different professionals: an atmosphere of buoyancy that lasted during the whole meeting, as emphasized in a previous study.^5^ Further, the forums exercised the concept of how the work of one member of a team would influence or connect with or influence the work of other members of the team in a mirroring experience, not in a top-down geometry but as an interrelated chain of network relationships and culture. The forums were also an important channel for informing the staff of the strategic institutional guidance from the highest level of governance of the hospital. By the virtual nature of the meetings, participants were able to be “on the same page”, without actually interfering with the ongoing activities of the operative theater or with everyday practice (e.g., surgical facilities, sterilizing material, catheterization laboratory, outpatient clinics). To be able to become conscious of the complexities of the connections and of the work was an upgrade for the staff. Interestingly, one of the key pillars of high-performance teams was reported to be the ability to engage in patterns of interaction with a high degree of connectivity, as demonstrated in a study of 60 teams (15 high performance team, 26 medium performance team, and 19 low performance team).^5^ The process the authors report started with what we called generative listening and mirroring. It was clear for us that the intangible dimension, related to the organizational culture, was a key issue to reach tangible performance improvement as previously suggested.^7^ By participating in the process as members of the local staff and not as outsiders, the authors followed a refined empiric observation-based measures,^8^ with the careful guidance of a proper management “style”.^9^ to stimulate engagement of the colleagues, to gradually build the experience in connection with the organizational culture, and following institutional guidance.

One of the key issues of our practice, the authors believe, was the periodicity of meetings and its duration. Empiric perceptions by us were that weekly meetings were convenient to keep the contextual connections alive and active; working forums were not permitted to last more than 60 minutes, even though stuff might require more time; in this case, contents were postponed to the next forum; occasionally, they lasted less than 60 minutes. The authors avoided working forums with frequency higher than a two-week interval, as was the case in the mandatory subcommittees of hospital regulation that the authors must comply with. Frequent interdisciplinary work made communications easier, clear, synthetic and fast (daily communication between the authors); participants felt safe and comfortable; preventing unnecessary or additional partial meetings of a few professionals that might not be representative and resolutive in the processes discussed; in addition, also hindered conflicts or unsafe feelings in the working scenarios.^10^ It was interesting that some controversial or sensitive issues were examined with an interdisciplinary focus without pointing fingers, without attributing blame, as declared by one of the participants of a study of a systemic view of processes.^3^ The regular rhythm and frequency of the sessions brought the interdisciplinary team to the same page, so that they naturally became used to examining together other issues of everyday practice frequently related to many different professionals. Being together, listening, discovering, understanding, thinking, and planning before immediate actions, surprisingly, often took just a few minutes. This approach was more efficient and rapid than multiple small talks between two professionals or in small meetings dissociated from the whole context, giving a soft character to the interactions.^3^ in the forums. Some findings were unexpected, serendipitous and gave rise to specific working group gains, such as sustainability.

Our experience agreed with the concept demonstrated in a study where thematic analysis resulted in a connection graph showing how the identified 37 statements were related to themes studied and how these statements and themes were interrelated. Overall, there were more statements attached to intangible themes than to tangible ones. Separately, themes covering most statements were psychological safety, leadership, organizational structures and processes, communication openness, and employee and job attributes.^7^

The management structure the authors devised was focused in organizational culture and important dimensions of practice that were considered to have links with adverse events, patient safety, professional well-being, competitive advantage, and organizational performance; it was emphasized that intangible dimensions were scarcely represented as key dimensions in organizational culture.^1^ The level of psychological safety was reported to be more important than the level of education or years of experience of team member;s.^9^ the importance of a dynamic balance between inquiry and advocacy, avoiding being stuck in either of them, was previously recognized.^5^ Interestingly, the kind of attractors varied between low (point attractors), medium (limit cycles) and high-performance teams (low-dimensional chaotic attractors).^5^ In the evaluation of thinking, it may be difficult to find the way out of point attractors and limit cycles; in limit cycles, the authors may become locked in the same repetitive cycle, impoverishing performance.^5^^,^^11^

The kind of care the authors deliver to the community – as a tertiary referral center for healthcare ‒ requires that we work as a high-performance team. It was previously quoted.^9^ that each member contributes to the team and the entire group might be responsible for the team’s success: high-performance teams were characterized by an atmosphere of buoyancy. By showing appreciation and encouragement to members in the team, high-performance teams create emotional spaces that are expansive and open possibilities for action and creativity. In addition, the team accomplishes their tasks with ease and grace. In stark contrast, low-performance teams struggle with their tasks, operate in very restrictive emotional spaces created by a lack of mutual support and enthusiasm, often in an atmosphere charged with distrust and cynicism, as previously emphasized.^5^ The work of the healthcare professionals may be considered demanding either from a humane perspective.^12^ or because of the refined technical skills required. To keep teams motivated and protected against burnout is one of the aims of a sustainable management system.^10^ and was one of the aims of the current system. In addition, the authors suppose that in a receptive environment, it would prevent the occurrence that team members becoming inadvertently accidental adversaries.^3^

In generative listening, the authors gave a high endorsement to spontaneous contributions that occurred by serendipity, as our colleagues were conjoined in a receptive and supportive environment, started by listening and professional proximity. The authors were particularly open to contributions of details usually not seen in the connection of processes, “unseen” (according to Ms. Marcia Takeiti, RN)* or “unnoticed”.^13^ that the authors regarded as unknown. The balance between other colleagues and self-orientation, perception of internal strengths and weakness, would make it possible to identify opportunities^5^ for better processes and care. Schematic representations such as flowcharts and top-down operational geometry usually are not able to demonstrate the time necessary for listening and constructing bridges of the human actors that make the work processes smooth. Contributions of listening frequently would be able to detect inconsistencies of human dimensions or connections of health care processes that would be able to be more efficient or more ergonomic.

By mirroring the authors worked an attenuated hierarchical organizational geometry by supporting the professionals in practice, in a network geometry, preventing from spontaneity of findings being not perceived and making possible that each specific action or procedure would be guided by the proper professional or leadership in the line of care. The authors appraised creating an emotional space receptive and open for effective action, avoiding getting stuck in a restrictive emotional space, as previously emphasized.^5^ The central position in the figure (Fig. 1B) is of patients, but during healthcare processes, other members of the team may be in the central position (physician, surgeon, nurse, physiotherapist, etc.) according to the needs of the patient and families.

In the generative sequence (Fig. 1C), the authors have been emphasizing that, in addition to technological resources in health care, the care of patients is rooted in the human skills of professionals to give proper support for patients, families, and also colleagues, so that the team might work as smoothly as possible. The circles are plenty of pores, so that each step may be sensitive to feedback of going back and forth, refreshing and constantly updating the needs of the processes.

In generative projects, looking for results and numbers that may demonstrate it, the authors devised a way to reach the practical implications of the foundations we have been working on. Each number may be an abstract concept in a formal series; they may be considered terms of an equation.^14^ The strategy the authors were working on may be considered a human equation of multiple relationships; hence, our emphasis is on the statistical analysis (usually complex functions) over time. The risk of being trapped in repetitive response cycles may lock perceptions and not contribute to staff upgrade or enrichment.^5^

### Limitations

One limitation of our work is that the authors report the construction of an intangible structuring of surpassing institutional segmentation and is respectful to local skills leadership. Other potential limitations of our work were the risk of meeting fatigue. Usually, colleagues participate in the forums once or twice a week; participants of a specific service would have one or two meetings each week, attenuating the risk of fatigue. Maybe some colleagues are not devoted to the clinical governance strategies the authors work on in the forums or dissent from this strategy; the authors respect it. Based on the hypothesis that non-participants might be dissenters, the number is small.

## Conclusion

The experience after studying local culture with the help of colleagues of the staff seized the opportunity to build strategies to surpass the potential less ergonomic or aspect of compartmentalization necessary in complex tasks or procedures. High value was placed on sharing experiences with colleagues and constructing bridges between the human dimension of the skilled health care professionals of the staff. The authors brought nearness to interdisciplinary teams to provide psychological safety, increase engagement, sense of belonging, and creativity to the point of identifying details of processes, some of them usually not exposed to view, to make proper diagnoses to action accordingly to institutional demands and best care of patients. The authors believe that on the basis that the present study they have been working on, connecting concrete questions of clinical practice in health care, they would be able to build sustainable trust in the skilled team that would make actions sustainable and generative over time for the institution and to the patients served, and more broadly to society that supports us.

## Authors’ contributions

Alfredo Jose Mansur: Conceptualization, methodology, investigation, writing original data, review, and editing.

Orival de Freitas Filho: Conceptualization, methodology, investigation, writing original data, and editing.

Cicero Piva de Albuquerque: Conceptualization, methodology, investigation, writing original data, review, and editing.

Brigitte Feiner de Mello: Methodology, validation, review, and editing.

Leila Suemi Harima Letaif: Methodology, validation, review, and editing.

Edivaldo Massazo Utiyama: Supervision, methodology, validation, investigation, review, and editing.

## Data availability statement

All data are available within the text and may be requested from the corresponding author.

## Declaration of competing interest

The authors declare no conflicts of interest.
